# Peninsular Journal of Medicine and the Collateral Sciences

**Published:** 1853-09

**Authors:** 


*Peninsular Journal of Medicine and the Collateral Sciences*, conducted by the Medical Faculty of the University of Michigan, edited by E. ANDREWS' A.M. M.D., Demonstrator of Anatomy in the University, Ann Arbor Mich. Issued monthly at $2 per annum, in advance.

WE have received the first and second numbers of our Peninsular neighbor. The original and selected matter, as well as the mechanical execution, is good, and we congratulate our Michigan friends on the favorable impression they must have made in their first introduction to their readers.

The editor, Dr. Andrews, is a strong, vigorous writer, of thorough literary and scientific attainments. We bespeak for him the kind regards of our editorial confreres, for we know him to be a good man and true. J.

